# Transcutaneous auricular vagus nerve stimulation modulates masseter muscle activity, pain perception, and anxiety levels in university students: a double-blind, randomized, controlled clinical trial

**DOI:** 10.3389/fnint.2024.1422312

**Published:** 2024-07-10

**Authors:** Luciano Maia Alves Ferreira, Ricardo Brites, Gonçalo Fraião, Gonçalo Pereira, Henrique Fernandes, José Américo Almeida de Brito, Laura Pereira Generoso, Maria Gabriela Maziero Capello, Gabrielly Santos Pereira, Robson Dias Scoz, Josie Resende Torres Silva, Marcelo Lourenço Silva

**Affiliations:** ^1^Neuromodulation and Pain Unit (NeuroPain), Egas Moniz Center for Interdisciplinary Research (CiiEM), Egas Moniz School of Health & Science, Almada, Portugal; ^2^Laboratory of Neuroscience, Neuromodulation and Study of Pain (LANNED), Federal University of Alfenas - UNIFAL-MG, Alfenas, Minas Gerais, Brazil

**Keywords:** transcutaneous auricular vagus nerve stimulation (TAVNS), anxiety, masseter muscle activation, pain, neuromodulation

## Abstract

**Introduction:**

Chronic anxiety is a statemarked by sustained activation of the masseter muscle, manifesting in both mental and physical strain. This prolonged tension can significantly impact mental wellbeing and cognitive abilities, posing a risk for a range of health complications. This double-blind, randomized, controlled clinical trial investigated the impact of transcutaneous auricular vagus nerve stimulation (TAVNS) on masseter muscle activity, pressure pain threshold (PPT), and anxiety levels in university students with elevated anxiety.

**Methods:**

Forty-two participants meeting inclusion criteria were randomly assigned to either active TAVNS or sham TAVNS groups. Various parameters, including masseter muscle electromyographic (EMG) signals, PPT, and Beck Anxiety Inventory (BAI) scores, were assessed before pretreatment, immediately after the intervention week, and 2 weeks follow-up.

**Results:**

Active TAVNS significantly reduced both left and right masseter activation during resting mandibular position, persisting for 2 weeks post-intervention. Additionally, TAVNS induced a lasting decrease in both left and right masseter PPT, indicative of altered pain perception. Notably, BAI scores showed a substantial reduction, emphasizing TAVNS as a potential intervention for anxiety, with effects maintained at the 2-week follow-up.

**Discussion:**

This study provides comprehensive insights into the multifaceted effects of TAVNS on physiological and psychological aspects associated with anxiety in university students. The promising results underscore TAVNS as a potential neuromodulatory intervention for anxiety-related conditions, warranting further research and clinical exploration.

**Clinical Trial Registration:**

https://ensaiosclinicos.gov.br/rg/RBR-4s4kt2r.

## 1 Introduction

According to the American Psychological Association (2022), anxiety is defined as the anticipation of a future threat, distinct from fear, which elicits an immediate real or perceived response that influences the cognitive aspects of apprehensive expectation (referred to as normal adaptive anxiety). University students face burdens and concerns that differ from other age and occupational groups. Although exciting, invigorating, and empowering, these can be stressful, leading to anxiety and various forms of psychopathology due to academic overload, constant pressure for success, competition with peers, lack of leisure time, and less time with family (Mikolajczyk et al., 2008; Crocq, 2015).

Anxiety can be considered an involuntary psychological response of the central nervous system, with the aim of preparing the body to react mentally and/or physically to a harmful situation. Anxiety can lead to changes in muscle tension, with an increase in the activities of the sympathetic and parasympathetic nervous systems (Fernandez Rojas et al., 2023).

When muscle tension is present, oxidative metabolism increases, leading to a decrease in adenosine triphosphate (ATP), adenosine diphosphate (ADP), and phosphocreatine levels. This energy decrease results in reduced oxygenation in muscle tissues and an increase in nociceptive (pain) activity, especially in type I fibers associated with static and postural tone (El Assar et al., 2022). Huguenin (2004) identified that increased muscle tension and fatigue can lead to tension points (muscle hyperactivity) found in tense bands of skeletal muscle.

Several studies have conducted analyses of muscle activation patterns using surface electromyography to determine the influence of anxiety on the masticatory muscles (Owczarek et al., 2020a). Others have reported that increased psychoemotional stress and higher anxiety levels are associated with increased muscle tone in the masticatory muscles of university students and that anxiety can alter electromyographic (EMG) recordings of muscle activity (Owczarek et al., 2020b; Szyszka-Sommerfeld et al., 2023).

Transcutaneous auricular vagus nerve stimulation (TAVNS) has gained increasing attention as a non-invasive approach to anxiety reduction. Several scientific studies support the efficacy of TAVNS in alleviating anxiety symptoms. A randomized controlled pilot study by Hein et al. (2013) demonstrated the potential of auricular transcutaneous electrical nerve stimulation in reducing anxiety in depressed patients. Additionally, research by Wang et al. (2023) delved into the broader applications of vagus nerve stimulation (VNS), emphasizing its role in modulating anxiety-related factors. Furthermore, studies such as Yakunina et al. (2017) have explored the optimization of TAVNS techniques, utilizing functional MRI to better understand its effects on anxiety and related neural pathways. These findings collectively underscore the promising utility of TAVNS as a non-pharmacological therapeutic strategy for anxiety management, providing a safe and effective alternative for individuals seeking anxiety relief.

Therefore, our study aims to investigate the effects of TAVNS on various physiological and psychological parameters among university students. Specifically, we hypothesize that TAVNS will lead to a reduction in anxiety levels, an increase in the pressure pain threshold (PPT), a decrease in electromyographic (EMG) hyperactivity of the masseter muscles, and modulation of bite force in the masticatory muscles. These hypotheses are grounded in previous research suggesting the potential anxiolytic and analgesic effects of vagus nerve stimulation, as well as its ability to modulate autonomic nervous system activity and muscle tension. By exploring these hypotheses, we aim to gain insights into the potential therapeutic benefits of TAVNS for addressing both psychological and physiological aspects of stress and musculoskeletal function in university students.

## 2 Methods and materials

### 2.1 Participants

This is a double-blind, randomized, controlled clinical trial. Ethical approval was granted by the Egas Moniz School of Health's Technical-Scientific Council (CTC) and Ethics Committee (CEEM). Prior to participation in the study, informed consent was obtained from all subjects and/or parents or legal guardians of the participants. The study focused on university students in health-related courses, with participants randomly assigned to experimental (G1-TAVNS) or sham (G2-Sham) groups. Both participants and researchers administering treatments were blinded to group allocation, with blinding concluding at the study's end. Inclusion criteria involved healthy individuals aged 18–25 with complete dentition and informed consent with BAI scores of 7 or higher (Bardhoshi et al., 2016), while exclusion criteria included various medical and lifestyle factors. Dependent variables included anxiety levels, pressure pain thresholds, EMG muscle activation in the masseter muscles, and bite force, while independent variables included TAVNS intervention and assessment time points (pretreatment, post-treatment, and follow-up). The study was conducted at the Physical and Functional Assessment Laboratory in Physiotherapy (LAFFFi) at the Egas Moniz Interdisciplinary Research Center (CiiEM). This study was approved by Egas Moniz (CAAE 51925921.9.0000.5142) and registered in the Brazilian Registry of Clinical Trials (ReBEC) number RBR- 4s4kt2r.

### 2.2 Instruments

#### 2.2.1 Beck Anxiety Inventory

According to Quintão et al. (2013), BAI is an anxiety measurement instrument that assesses the neurovegetative components of anxious responses and combines the evaluation of physiological components with cognitive and behavioral components. It is one of the most commonly used clinical assessment scales. The BAI consists of 21 questions describing anxiety symptoms, and participants are needed to self-assess. Each question is scored from 0 (not at all) to 3 (severely). The scale has a total score of 63 points. To date, 11 studies have explored the diagnostic validity of BAI, with optimal cutoff scores ranging from 7 to 26 depending on the diversity of studied samples (Bardhoshi et al., 2016).

#### 2.2.2 Pressure pain threshold

PPT was assessed using a portable pressure algometer with a 1 cm^2^ rubber tip, featuring a digital communication interface and applied perpendicularly to the skin at a rate of 0–50 kgf/s (PHYSIOCODE, Belo Horizonte, MG, Brazil). PPT was defined as the minimum pressure evoking the first painful sensation. PPT was measured in the masseter muscles three times with a 10-s interval for each point, and the average value was used for statistical analysis (Ylinen et al., 2003).

#### 2.2.3 Electromyography and dynamometry

For bilateral masseter electromyographic recording, a 12-channel biological signal acquisition system model SAS2000V4-WiFi with an integrated bite dynamometer (EMG System do Brazil, São José dos Campos, SP, Brazil) was used. Disposable circular Kendall electrodes measuring 30 × 24 mm (MEDITRACE-−100, São Paulo, Brazil) were employed for surface EMG signal recording, positioned according to the guidelines of the Surface Electromyography for the Non-Invasive Assessment of Muscles (Stegeman et al., 2000). The skin was cleaned with 70% ethyl alcohol to reduce impedance.

The processing of the EMG signals involved full-wave rectification, linear enveloping using a fourth-order Butterworth filter with a cut-off frequency of 5 Hz, and normalization in both time and amplitude. The amplitude was normalized based on the mean amplitude of the full-wave rectified EMG signal (Drost et al., 2006). The root mean square (RMS) value was used to assess the signal amplitude.

### 2.3 Interventions

#### 2.3.1 TAVNS stimulation

For TAVNS, the DS100CBM electrostimulator (SIKURO, Niteroi, RJ, Brazil) was used with cylindrical Clip Ear electrodes (NKL, Brusque, SC, Brazil). The technique applicator received the device, which was programmed by the supervisor in a blinded manner regarding the equipment settings and the type of stimulus to be administered (real or sham). For active TAVNS stimulation, the current level used during stimulation was set to 0.5 mA. The waveform was biphasic, which helps reduce potential tissue damage and provide a balanced charge over the stimulation period. The work cycle consisted of continuous 25 Hz stimulation for 20 min per session, administered on three alternate days. The micropulsation period was set to 500 μs, which has been shown in previous research to be effective for taVNS (Busch et al., 2013). The exact location of the stimulation was the left cymba conchae, a region innervated by the auricular branch of the vagus nerve, ensuring targeted and effective vagal modulation. For sham TAVNS, the technique was conducted in the same manner as active stimulation, but the device was programmed by a research assistant to provide 0 Hz, indicating no actual stimulation was delivered. This programming was done without the knowledge of the responsible investigator and the technique applicator, maintaining the study's blinding. Patients were informed that they might not feel the current during the intervention due to its lower intensity, without disclosing the true nature of the sham stimulation. Thus, the sham group did not receive effective stimulation, addressing concerns about potential bias in placebo participants.

### 2.4 Experimental procedures

**Figure 1:**
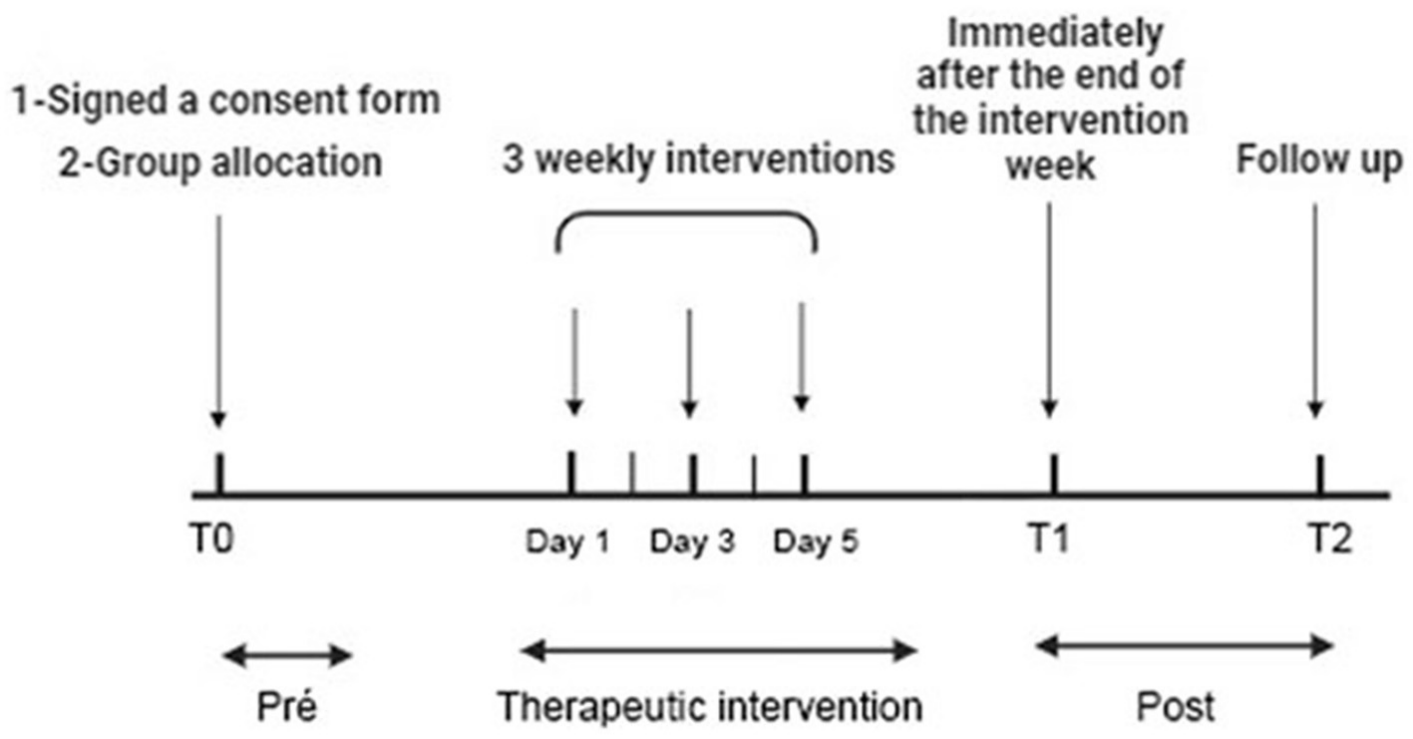
Experimental procedure with time course. Participants selected based on criteria completed forms and consents. Beck Anxiety Inventory, EMG, and algometry assessed anxiety. EMG recorded resting and contraction signals from masseter muscles. Pressure pain threshold (PPT) followed. True and sham TAVNS were applied in 20-min sessions on alternate days for a week. Data collected pre (T0), post (T1), and 2 weeks post-intervention (T2) per CONSORT/2010 guidelines.

The procedures encompassed several steps (Figure 1): participants were selected based on inclusion and exclusion criteria through the completion of a Sample Characterization Form, followed by the signing of Informed Consent Forms. On assessment days, participants completed the Beck Anxiety Inventory in ~5 min, followed by an explanation of the procedures to be administered, including electromyography and algometry. The masseter muscle area was cleaned with 70% ethyl alcohol to reduce impedance, and electrodes were symmetrically positioned on both sides, covering the target muscles. EMG assessments were conducted in two phases, recording EMG signals during the resting mandibular position (rest EMG) and after three maximum voluntary contraction (MVC) against a bite dynamometer, with each signal sample lasting 10 s. PPT followed EMG assessments, applying pressure to the specified muscle points. Active TAVNS stimulation (TAVNS Group), or sham TAVNS was administered on alternate days over the course of 1 week (a total of 3 weekly interventions). Data related to the study were collected at three distinct time points: T0 before the intervention (Pre), T1 immediately after the end of the intervention week (Post), and T2 2 weeks after the end of the intervention (Follow-Up; FU), following CONSORT/2010 guidelines.

### 2.5 Potential side effects

Potential side effects of the procedure are minimal, and there have been no reports of significant adverse events associated with low-current procedures similar to those used in this study. For TAVNS the possible side effects may include ear pain, dizziness, skin redness, and headache (Kim et al., 2022). If any of these side effects occur, the participant will be closely monitored. If the symptoms persist for more than 1 h, the participant will be referred to a medical professional for further evaluation. Instances of discontinuation or withdrawal from the study will be recorded in the study database.

### 2.6 Statistical analysis

For statistical analysis, the Statistical Package for the Social Sciences (SPSS) software (IBM Corp., Chicago, USA), version 20.0, was used. Initially, all data sets from the sample were tested for normality using the Kolmogorov-Smirnov test. After this analysis, a two-way analysis of variance (ANOVA) test was conducted if the sample followed a normal distribution. If the data did not meet the normality assumption, the Kruskal-Wallis test was applied. Regarding the independent variables, if the normality criterion was met, a Student's *t*-test was conducted. If the criterion was not met, the Mann-Whitney *U*-test was used for intergroup comparisons.

To examine the relationships between anxiety levels, resting masseter muscle activity, and PPT, Pearson's correlation coefficient was used. Correlation coefficients were interpreted as follows: small (0.1–0.3), medium (0.3–0.5), and large (0.5–1.0) based on Cohen's guidelines. A *p*-value of < 0.05 was considered statistically significant.

## 3 Results

**Figure 2:**
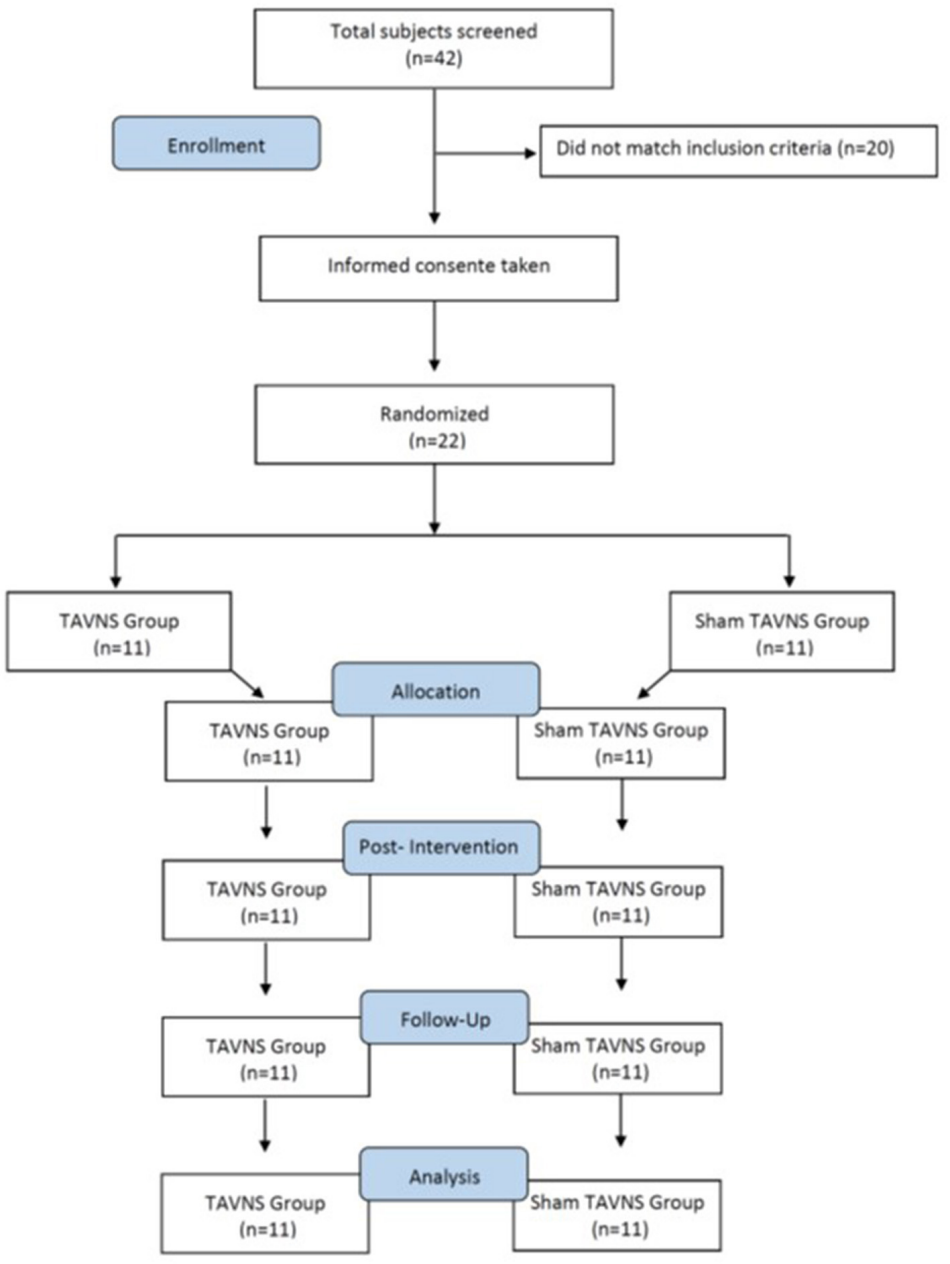
Participant selection and characteristics (CONSORT). A total of 42 participants responded, with 22 meeting inclusion criteria (BAI scores ≥7) randomly assigned to TAVNS and sham TAVNS groups (*n* = 11 each). Participants aged 18–25, comprised 58.7% females and 41.3% males. EMG activity was detectable in all, with no side effects during measurements or TAVNS. All parameter data exhibited normal distribution.

A total of 42 participants responded to this study. After applying the inclusion criteria, 22 individuals who presented BAI scores of 7 or higher were randomly divided into our active or sham TAVNS stimulation groups (*n* = 11; CONSORT, Figure 2). The age was between 13 years old (48.3%) and 14 years old (51.7%); 58.7% (*n* = 193) were female, and 41.3% (*n* = 136) were male. EMG activity of the masseter muscle was detectable in all participants and with both EMG measures at rest and MVC. There were no side effects or undesired events during measurements and TAVNS stimulation. Data for all parameters were normally distributed. The authors conducted a comprehensive examination of the dataset to identify outlier participants across the variables under investigation. However, no outliers were found upon thorough analysis.

### 3.1 Changes in masseter EMG signals during the resting mandibular position

**Figure 3:**
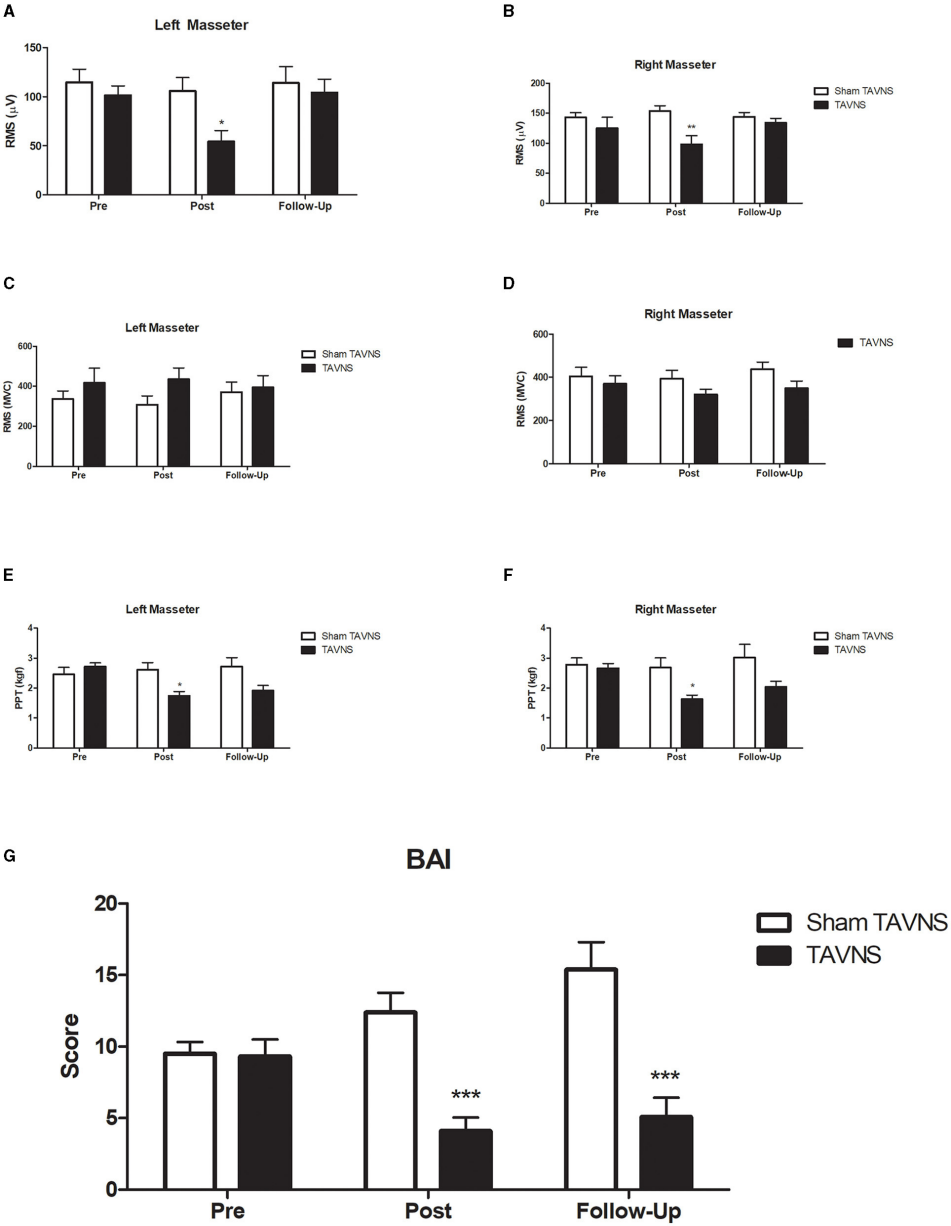
Experimental results. Transcutaneous auricular vagus nerve stimulation (TAVNS) on masseter electromyography (EMG) signals and pressure pain threshold (PPT) in participants with elevated Beck Anxiety Inventory (BAI) scores. TAVNS significantly reduced left **(A)** and right **(B)** masseter activation during rest, recovering to baseline after 2 weeks, with a distinct post-treatment difference between groups. However, TAVNS had no effect on masseter activation during maximum voluntary contraction [MVC; **(C)** left masseter muscle; **(D)** right masseter muscle]. Notably, TAVNS reduced both left **(E)** and right **(F)** masseter PPT, with recovery observed after 2 weeks, while sham TAVNS had no impact on PPT. Furthermore, TAVNS exhibited a significant and lasting reduction in BAI scores **(G)**, emphasizing its potential as an effective intervention for anxiety. *p < 0.05; **p < 0.01; ***p < 0.001.

Three sessions of TAVNS stimulation reduced left masseter activation during rest from an average ratio of 101.28 in the pretreatment period to 54.32 in the post-treatment period (*p* < 0.05) and returned to basal values after 2 weeks of follow-up (104.20; *p* > 0.05; Figure 3A). The resting EMG data for the left masseter muscle showed a significant difference between the groups at T1 (*p* < 0.05). The data yielded a significant time effect [*F*_(2, 40)_ = 5.652, *p* = 0.009] but not at treatment [*F*_(1, 20)_ = 2.834, *p* = 0.107) or time × treatment interaction [*F*_(2, 40)_ = 2.788, *p* = 0.0735]. The same activation pattern was found for the right masseter during rest from an average ratio of 124.90–98.25 (*p* < 0.05) and returned to basal values after 2 weeks of follow-up (134.39; *p* > 0.05; Figure 3B), showing a significant difference between groups at post-treatment (*p* < 0.05). The resting right masseter EMG data yielded a non-significant time effect [*F*_(2, 40)_ = 0.632, *p* = 0.536] or time × treatment interaction [*F*_(2, 40)_ = 2.028, *p* = 0.1450] but had a significant treatment effect [*F*_(1, 20)_ = 10.570, *p* = 0.0040]. Sham TAVNS stimulation did not change left or right masseter activation during rest in the pretreatment, post-treatment, or follow-up phases.

### 3.2 Changes in masseter EMG signals during maximum voluntary contraction

TAVNS or Sham TAVNS stimulation did not change left or right masseter activation during MVC in the pretreatment, post-treatment, or follow-up phases (Figures 3C, D). Normality tests confirmed that the data for this analysis were normally distributed (Shapiro-Wilk test, *p* > 0.05). For the left masseter (Figure 3C), no significant interaction was detected between the time effect [*F*_(2, 40)_ = 0.1190, *p* = 0.8881], treatment [*F*_(1, 20)_ = 1.151, *p* = 0.2960], or time × treatment interaction [*F*_(2, 40)_ = 2.363, *p* = 0.1071]. For the right masseter (Figure 3D), no significant interaction was detected between the time effect [*F*_(2, 40)_ = 1.668, *p* = 0.1986], treatment [*F*_(1, 20)_ = 2.025, *p* = 0.1666], or time × treatment interaction [*F*_(2, 40)_ = 0.8038, *p* = 0.4531].

### 3.3 Changes in masseter PPT

Three sessions of active TAVNS stimulation reduced left masseter PPT from an average ratio of 2.71 in the pretreatment to 1.73 in the post-treatment (*p* < 0.05) and returned to basal values after 2 weeks of follow-up (1.91; *p* > 0.05, η^2^ = 0.28; Figure 3E), showing a significant difference between groups post-treatment (*p* < 0.05). Normality tests confirmed that the data for this analysis were normally distributed (Shapiro-Wilk test, *p* > 0.05). The data yielded a significant time effect [*F*_(2, 42)_ = 4.762, *p* = 0.0137], treatment [*F*_(1, 21)_ = 3.742, *p* = 0.0667] and time × treatment interaction [*F*_(2, 42)_ = 11.26, *p* = 0.0001]. Sham TAVNS stimulation did not change the left masseter PPT in the pretreatment, post-treatment, or follow-up phases. A similar activation pattern was found for the right masseter PPT from an average ratio of 2.65–1.63 (*p* < 0.05) and returned to basal values after 2 weeks of follow-up (2.04; *p* > 0.05; Figure 3F), showing a significant difference between groups post-treatment (*p* < 0.05). Right masseter PPT data yielded a significant time effect [*F*_(2, 42)_ = 6.830, *p* = 0.0027], treatment effect [*F*_(1, 21)_ = 5.110, *p* = 0.00345], and time × treatment interaction [*F*_(2, 42)_ = 5.649, *p* = 0.0067]. Sham TAVNS stimulation did not change the right masseter PPT in the pretreatment, post-treatment, or follow-up phases.

### 3.4 TAVNS changes in BAI scores

Three sessions of active TAVNS stimulation reduced the left masseter PPT from an average ratio of 9.3 in the pretreatment phase to 4.1 in the post-treatment phase (*p* < 0.05) and was maintained after 2 weeks of follow-up (5.1; *p* < 0.05; Figure 3G), showing a significant difference between groups in the post-treatment and follow-up phases (*p* < 0.05). Normality tests indicated that the data for this analysis were normally distributed (Shapiro-Wilk test, *p* > 0.05). The data yielded a significant treatment effect [*F*_(1, 18)_ = 18.60, *p* = 0.0004] or time × treatment interaction [*F*_(2, 36)_ = 15.01, *p* = 0.0001] but not a time effect [*F*_(2, 36)_ = 2.115, *p* = 0.1354]. Sham TAVNS stimulation did not change BAI in the pre-treatment, post-treatment, or follow-up phases.

### 3.5 Correlation between anxiety levels, masseter muscle activity, and pressure pain threshold

We analyzed the correlation between BAI scores and resting masseter muscle activity, as well as PPT using Pearson's correlation coefficient. We found significant correlations between these variables, indicating a link between anxiety levels and both muscle activity and pain perception. Specifically, higher BAI scores were associated with increased resting masseter muscle EMG activity (*r* = 0.54, *p* < 0.01) and decreased PPT (*r* = −0.47, *p* < 0.01), suggesting that elevated anxiety levels correspond to heightened muscle tension and lower pain tolerance.

### 3.6 Adverse events

In the course of this study, no adverse events were reported by any of the participants. All individuals who underwent both active and sham TAVNS sessions tolerated the procedures well. No participants experienced discomfort, skin irritation, or any other negative side effects related to the stimulation. The absence of adverse events supports the safety and feasibility of TAVNS as a non-invasive intervention for anxiety, pain perception, and muscle activity modulation in university students.

## 4 Discussion

In this study, we investigated the effects of TAVNS on masseter muscle EMG signals and PPT and BAI scores in volunteers with elevated anxiety levels. A total of 42 participants were included, and 22 individuals with BAI scores of 7 or higher were randomly assigned to either active TAVNS or sham TAVNS stimulation groups. The data showed that three sessions of TAVNS significantly reduced left and right masseter activation during rest, returning to baseline after 2 weeks. Additionally, TAVNS led to a significant decrease in both left and right masseter PPT, indicative of altered pain perception. Notably, active TAVNS demonstrated a substantial reduction in BAI scores, emphasizing its potential as a non-invasive intervention for anxiety with persistent effects observed at the 2-week follow-up. Sham TAVNS, in contrast, did not elicit similar changes in any of the parameters measured. These findings underscore the promising therapeutic potential of TAVNS in modulating both physiological and psychological aspects associated with anxiety.

The analysis of masseter EMG signals during the resting mandibular position revealed significant reductions in both left and right masseter activation following three sessions of active TAVNS. The reduction persisted until the 2-week follow-up, indicating a lasting effect on muscle activity. The absence of significant changes in the sham TAVNS group suggests the specificity of the observed effects to active TAVNS stimulation. Interestingly, alterations in EMG signals were not observed during MVC, indicating that TAVNS selectively influences resting muscle activity without affecting muscle engagement during voluntary contraction.

Participants were instructed to keep their masseter muscles at rest by sitting comfortably and maintaining a relaxed jaw position without clenching their teeth. Rest was defined as a state where the participants' jaw muscles were not engaged in any active movement or forceful contraction. The muscle activity recorded at rest, which was about 50–100 μV is not noise but indicative of the baseline muscle tone (Botelho et al., 2011). This baseline activity is consistent with other studies that report similar levels of EMG activity in resting muscles (Szyszka-Sommerfeld et al., 2020). In individuals with high levels of anxiety, the masseter muscle activity at rest is often elevated due to increased muscle tension, which can result in higher EMG readings compared to less anxious individuals (Zieliński et al., 2021).

The observed reduction in masseter EMG signals during the resting mandibular position following active TAVNS aligns with previous studies indicating the modulatory effects of VNS on muscle activity (Redgrave et al., 2018; Badran et al., 2020). Interestingly, the lack of influence on EMG signals during maximum voluntary contraction suggests a selective impact on resting muscle tone. This finding corroborates the specificity of TAVNS effects on muscle activity during relaxed states, potentially mediated by the vagal regulation of autonomic functions (Kong et al., 2018).

Moreover, PPT assessments demonstrated that TAVNS led to a significant reduction in both left and right masseter PPT, reflecting altered pain perception in response to stimulation. The effects were sustained until the 2-week follow-up, underscoring the durability of TAVNS-induced changes in pain sensitivity. Notably, sham TAVNS did not produce similar effects, highlighting the specificity of the observed alterations to active TAVNS stimulation.

We found that elevated anxiety levels correspond to heightened muscle tension and lower pain tolerance. These findings are consistent with existing literature, which highlights the impact of anxiety on muscle activation and pain sensitivity (Burston et al., 2019; Zetterman et al., 2021). The physiological mechanisms underlying these associations likely involve the autonomic nervous system's response to stress, wherein heightened anxiety can lead to increased sympathetic activity, resulting in greater muscle tension and altered pain perception (Bali and Jaggi, 2015). By demonstrating these correlations, our study underscores the interconnectedness of psychological and physiological parameters in the context of anxiety and supports the therapeutic potential of TAVNS in addressing these multifaceted aspects.

The relationship between PPT and anxiety levels is complex, as increased anxiety has been shown to correlate with heightened pain sensitivity, resulting in lower PPT values (Liao et al., 2023). Typically, individuals without high levels of anxiety exhibit higher PPT values, indicating lower pain sensitivity. A decrease in PPT could indeed signal increased hypersensitivity, particularly against a backdrop of elevated anxiety levels. TAVNS can modulate pain perception through its influence on the autonomic nervous system and central pain pathways. By stimulating the vagus nerve, TAVNS activates the descending pain inhibitory pathways and modulates the release of neurotransmitters such as serotonin and norepinephrine, which are involved in pain modulation (Napadow et al., 2012). This mechanism helps in reducing pain sensitivity and increasing PPT, highlighting the therapeutic potential of TAVNS in managing anxiety-related pain hypersensitivity.

The alterations in PPT, indicative of changes in pain perception, are consistent with studies demonstrating the analgesic effects of TAVNS (Napadow et al., 2012; Frangos et al., 2017). The sustained reduction in PPT after 2 weeks suggests a lasting impact on pain sensitivity, supporting the potential role of TAVNS in modulating pain pathways. The lack of significant changes in the sham TAVNS group reinforces the specificity of these effects to active TAVNS stimulation.

In terms of anxiety levels assessed by the BAI, active TAVNS resulted in a substantial reduction in anxiety scores. This effect was maintained at the 2-week follow-up, indicating a lasting impact on anxiety symptoms. The absence of significant changes in the sham TAVNS group emphasizes the specificity of the observed improvements in anxiety to active TAVNS stimulation. The findings suggest a potential link between the modulation of masseter muscle activity, pain sensitivity, and anxiety levels, providing valuable insights into the multifaceted effects of TAVNS on both physiological and psychological aspects in adolescents with elevated anxiety.

The significant reduction in anxiety levels, as measured by the Beck Anxiety Inventory (BAI), following TAVNS is in line with research highlighting the anti-anxiety properties of VNS (George et al., 2008; Burger et al., 2016). The maintenance of these improvements at the 2-week follow-up suggests enduring benefits, emphasizing the potential of TAVNS as a sustained intervention for anxiety disorders in adolescents.

While significant improvements were observed in the active TAVNS group, it is pertinent to investigate whether BAI scores increased for sham subjects and the potential factors contributing to such changes. The study aimed to assess the impact of TAVNS on anxiety levels, pain perception, bite force, and masseter muscle EMG activation in university students before and after the final assessments of the academic year. Previous research suggests that factors such as stressors related to academic performance, social pressures, and the anticipation of future challenges could lead to heightened anxiety levels in university students (Bayram and Bilgel, 2008; Kristensen et al., 2023).

The observed reductions in masseter muscle EMG signals and improvements in anxiety levels following active TAVNS stimulation align with previous research elucidating the therapeutic mechanisms of TAVNS. Vagus nerve stimulation has been shown to modulate autonomic functions and emotional responses through its bidirectional communication with the central nervous system (Tracey, 2002). Specifically, TAVNS has been proposed to exert its effects by activating the cholinergic anti-inflammatory pathway, leading to the release of neurotransmitters such as acetylcholine and noradrenaline, which regulate autonomic tone and emotional states (Kox et al., 2014). Furthermore, TAVNS has been demonstrated to influence cortical and subcortical brain regions implicated in anxiety regulation, such as the amygdala and prefrontal cortex (He et al., 2013). These neurophysiological effects may underlie the observed reductions in masseter muscle activity and anxiety levels, highlighting the potential of TAVNS as a neuromodulatory intervention for anxiety-related conditions.

Additionally, the positive outcomes observed in this study may be attributed to the role of TAVNS in modulating the autonomic nervous system and its influence on neuroplasticity. Vagus nerve stimulation has been shown to enhance neuroplasticity by promoting the release of neurotrophic factors such as brain-derived neurotrophic factor (BDNF), which facilitate synaptic plasticity and neuronal regeneration (Roosevelt et al., 2006). Moreover, TAVNS has been associated with increased parasympathetic activity and reduced sympathetic activity, leading to improvements in heart rate variability and stress response regulation (Geng et al., 2022). These neurophysiological mechanisms may contribute to the observed reductions in masseter muscle activity and anxiety levels, highlighting the multifaceted effects of TAVNS on both physiological and psychological parameters.

Moreover, the analgesic effects of TAVNS observed in this study are consistent with previous findings demonstrating the modulation of pain pathways by vagus nerve stimulation. Vagus nerve stimulation has been shown to attenuate pain perception through its interaction with descending pain inhibitory pathways and the modulation of neurotransmitter release in pain-processing regions of the brain (Napadow et al., 2012; Frangos et al., 2015). The sustained reduction in pain sensitivity following TAVNS suggests enduring changes in pain processing, which may contribute to the overall therapeutic effects of TAVNS on anxiety symptoms. These findings highlight the complex interplay between autonomic regulation, emotional processing, and pain perception in the therapeutic mechanisms of TAVNS.

This study aligns with literature highlighting the bidirectional communication between the vagus nerve and the central nervous system, influencing both autonomic and emotional responses (Tracey, 2002; Kox et al., 2014). The utilization of TAVNS, a non-invasive and well-tolerated stimulation method, adds to the emerging body of evidence supporting its potential therapeutic applications in anxiety-related conditions (He et al., 2013; Yakunina et al., 2017).

It is essential to acknowledge potential biases that may influence the interpretation of the study's findings. First, the relatively small sample size, particularly in the subgroup analysis of active TAVNS and sham TAVNS groups, may limit the generalizability of the results. The random assignment of participants to these groups helps mitigate selection bias, but the possibility of unmeasured confounders influencing the outcomes cannot be entirely excluded. Moreover, the reliance on self-reported anxiety levels using the BAI introduces the potential for response bias, as participants may have altered their responses based on their awareness of the intervention. To address these concerns, future studies with larger sample sizes, double-blinded designs, and objective measures for anxiety assessment could provide a more robust understanding of the effects of TAVNS on masseter muscle activity, pain perception, and anxiety levels in adolescents.

## 5 Conclusion

In conclusion, this study contributes valuable insights into the multifaceted effects of TAVNS on both physiological and psychological aspects associated with anxiety in adolescents. The findings underscore the potential of TAVNS as a non-invasive neuromodulatory intervention, opening avenues for further research and clinical applications in the management of anxiety-related conditions in this population.

## Data availability statement

The original contributions presented in the study are included in the article/supplementary material, further inquiries can be directed to the corresponding author.

## Ethics statement

The studies involving humans were approved by Egas Moniz (CAAE 51925921.9.0000.5142) and registered in the Brazilian Registry of Clinical Trials (ReBEC) number RBR- 4s4kt2r. The studies were conducted in accordance with the local legislation and institutional requirements. The participants provided their written informed consent to participate in this study.

## Author contributions

LF: Conceptualization, Data curation, Formal analysis, Funding acquisition, Investigation, Methodology, Project administration, Resources, Software, Supervision, Validation, Visualization, Writing – original draft, Writing – review & editing. RB: Conceptualization, Data curation, Formal analysis, Funding acquisition, Investigation, Methodology, Project administration, Resources, Software, Supervision, Validation, Visualization, Writing – review & editing. GF: Conceptualization, Data curation, Formal analysis, Funding acquisition, Investigation, Methodology, Project administration, Resources, Software, Supervision, Validation, Visualization, Writing – review & editing. GP: Conceptualization, Data curation, Formal analysis, Funding acquisition, Investigation, Methodology, Project administration, Resources, Software, Supervision, Validation, Visualization, Writing – review & editing. HF: Conceptualization, Data curation, Formal analysis, Funding acquisition, Investigation, Methodology, Project administration, Resources, Software, Supervision, Validation, Visualization, Writing – review & editing. JB: Conceptualization, Data curation, Formal analysis, Funding acquisition, Investigation, Methodology, Project administration, Resources, Software, Supervision, Validation, Visualization, Writing – review & editing. LP: Conceptualization, Data curation, Formal analysis, Funding acquisition, Investigation, Methodology, Project administration, Resources, Software, Supervision, Validation, Visualization, Writing – review & editing. MM: Conceptualization, Data curation, Formal analysis, Funding acquisition, Investigation, Methodology, Project administration, Resources, Software, Supervision, Validation, Visualization, Writing – review & editing. GSP: Conceptualization, Data curation, Formal analysis, Funding acquisition, Investigation, Methodology, Project administration, Resources, Software, Supervision, Validation, Visualization, Writing – review & editing. RS: Conceptualization, Data curation, Formal analysis, Funding acquisition, Investigation, Methodology, Project administration, Resources, Software, Supervision, Validation, Visualization, Writing – review & editing. JS: Conceptualization, Data curation, Formal analysis, Funding acquisition, Investigation, Methodology, Project administration, Resources, Software, Supervision, Validation, Visualization, Writing – original draft, Writing – review & editing. MS: Conceptualization, Data curation, Formal analysis, Funding acquisition, Investigation, Methodology, Project administration, Resources, Software, Supervision, Validation, Visualization, Writing – original draft, Writing – review & editing.
